# Diplura and Protura of Canada

**DOI:** 10.3897/zookeys.819.25238

**Published:** 2019-01-24

**Authors:** Derek S. Sikes

**Affiliations:** 1 University of Alaska Museum, University of Alaska Fairbanks, Fairbanks, Alaska 99775-6960, USA University of Alaska Fairbanks Fairbanks United States of America

**Keywords:** biodiversity assessment, Biota of Canada, Diplura, Protura

## Abstract

A literature review of the Diplura and Protura of Canada is presented. Canada has six Diplura species documented and an estimated minimum 10–12 remaining to be documented. The Protura fauna is equally poorly known, with nine documented species and a conservatively estimated ten undocumented. Only six and three Barcode Index Numbers are available for Canadian specimens of Diplura and Protura, respectively.

Diplura, sometimes referred to as two-pronged bristletails, and Protura, sometimes called coneheads, are terrestrial arthropod taxa that have suffered from lack of scientific attention in Canada as well as globally. As both groups are undersampled and understudied in Canada, the state of knowledge is considered to be poor, although there have been some modest advances since 1979. Both of these taxa are soil dwelling, and, given the repeated glaciations over most of Canada, the Canadian diversity is expected to be relatively low except possibly in unglaciated areas. Nonetheless, the vast majority of the country has been poorly sampled which leaves boundless opportunities for those who develop an interest in these fascinating animals.

## Diplura

There are around 800 species of Diplura known worldwide (Chapman 2009) and approximately 170 species in North America (Allen 2002). Tomlin (1979a) reported there were no published records of Diplura in Canada; however, as unidentified specimens of the families Campodeidae and Japygidae were known from Canada, based on specimens in public and private collections, he recorded two species, presumably one from each family, from the country in 1979. Overlooked by Tomlin were at least four species that had been documented from Canada before 1979, two campodeids, *Haplocampadrakei* Silvestri and *Tricamparileyi* (Silvestri), and two japygids, *Occasjapyxamericanus* (MacGillivary) and *Evalljapyxsaundersi* Pagés (Silvestri 1933, 1948, Saunders 1946, Condé 1973, Reddell 1983, Pagés 1996). More recently, Alberto Sendra identified two additional campodeid species from Ontario, *Campodeafragilis* Meinert and *Campodeaplusiochaeta* (Silvestri), based on DNA barcoded Canadian specimens in the Barcode of Life Data System (BOLD; Ratnasingham and Hebert 2007). These species are known from US states bordering Canada (Allen 2002) and bring the total to six identified species for Canada.

To estimate the size of the complete fauna, I evaluated reports of incompletely identified Canadian dipluran specimens, DNA barcoded Canadian specimens in BOLD, and species known from near the Canadian border, but not yet recorded from Canada. Subsequent to Tomlin’s (1979a) report, Tomlin and Nagy (1979) reported a japygoid from Ontario that they thought was a species close to or presumably in *Parajapyx*. There continues to be uncertainty about the identity of this species. A *Parajapyx* is also said to occur in British Columbia (Cannings and Scudder 2006), but is most likely a misidentification of *Evalljapyxsaundersi* Pagés. Additionally, there are seven DNA barcoded specimens identified as *Tricamparileyi* which fall into two Barcode Index Numbers (BINs) that are each other’s nearest neighbors but are over 12% divergent (BIN BOLD:ACK8620, from Waterton Lakes National Park, Alberta; and BIN BOLD:ACX3814 known from Darkwoods Conservation Area and Glacier National Park, British Columbia). The very large genetic distance between these two BINs suggests they may correspond to different species. This could be the result of misidentification(s), lab mix-up, cryptic species (taxonomic undersplitting) or unusually high within-species genetic diversity. At least one specimen in each BIN was identified by Alberto Sendra, a taxonomist of Diplura, with the remainder identified by their DNA barcodes. For counting purposes, I refer to one of these BINs, which could otherwise be an unaccounted-for Canadian dipluran species, as Tricampacf.rileyi.

Two BINs of Canadian diplurans remain unidentified below the family Campodeidae (BOLD:AAN6530, BOLD:ACZ3071). The first of these BINs may correspond to *Haplocampadrakei*, already known from Canada, because it corresponds to specimens collected from 1074m in Jasper National Park, Alberta, and *H.drakei* is known from Banff, Alberta, so this is a possible match. The second BIN corresponds to a specimen from Toronto, Ontario and likely represents one of the eight campodeid species that Allen (2002) reports but have yet to be recorded from Canada, although they are known from states bordering Canada.

Sikes and Allen (2016) reported the northern-most records for Diplura in North America based on Alaskan specimens of *Metriocampaallocerca* Conde & Geeraert, which was described from northwestern Montana (Allen 2002), so it may occur in Canada.

To summarize the Canadian Diplura fauna, there are six species identified and 10–12 additional species expected to occur based on six BINs of DNA barcoded specimens, incompletely identified Canadian specimens, and species known from near the Canadian border. Thus, the Canadian dipluran fauna could be as high as 18 species, making 40–66% of this fauna undocumented. Although considerable progress has been made relative to the report of Tomlin (1979a), much of this has been based on discovery of overlooked literature, and clearly much work remains to be done to fully document the Canadian fauna.

**Table 1.** Census of Diplura in Canada.

Taxon^1^	No. species reported in Tomlin (1979a)	No. species currently known from Canada	No. BINs^2^ available for Canadian species	Est. no. undescribed or unrecorded species in Canada	General distribution by ecozone^3^	Information sources
**Suborder Rhabdura**						
Campodeidae	1	4	6	9–11	Mixedwood Plains, Montane Cordillera, Boreal Plains	Allen 2002; BOLD
**Suborder Dicellurata**						
Japygidae	1	2	0	0	Pacific Maritime, Montane Cordillera	Pagés 1996, Allen 2002
Parajapygidae	0	0	0	1	Hudson Plains	Tomlin and Nagy 1979
**Total**	**2**	**6**	**6**	**10–12**		

**^1^**Classification follows that of Allen (2002). **^2^**Barcode Index Number, as defined in Ratnasingham and Hebert (2013). **^3^**See figure 1 in Langor (2019) for a map of ecozones.

## Protura

Despite work by many eminent entomologists since the discovery of Protura in 1907, much remains unknown about these organisms (Pass and Szucsich 2011). Szeptycki (2002) estimated that only 10% of the world’s species have been described. Only 743 species are known worldwide (Szeptycki 2007), and there are approximately 92 species described from North America (Allen 2006, Szeptycki 2007). Tomlin (1979b) reported three species of Protura known from Canada, two species of Acerentomidae: *Verrucoentomoncanadense* (Tuxen) and *Vesiculentomoncondei* (Tuxen) (Shrubovych et al. 2014), both known from the unglaciated Richardson Mountains of the Yukon and known only from Canada, where they were described in 1955, and one unidentified eosentomid: *Eosentomon* sp.

Overlooked by Tomlin (1979b) were at least three additional species that had been described from Canada before 1979 by Rusek (1974): *Nippoentomonbifidum*, and *Nippoentomonkevani*, both known only from their type locality of Vancouver, British Columbia (BC), and *Vesiculentomonmarshalli* Rusek, known only from its type locality of Victoria, BC.

Since Tomlin’s summary (1979b), two additional species were described from Canada by Nosek (1984), *Eosentomonbernardi* and *Eosentomoncanadense*, both known only from their type localities of Ste-Anne-de-Bellevue, Québec, and one widespread species, *Acerentuloidesamericanus* (Ewing), reported from Québec by Nosek and Kevan (1984), but also known from several states of the USA. Behan-Pelletier (1993) listed nine species of Protura from Canada, one of which, *Acerentomon* sp., appears to be unique to her list, but no Nearctic species in this genus is listed in Szeptycki (2007) so this species cannot be reconciled, leaving eight identified Canadian species known as of 1993.

Bernard and Guzowski (2002) described *Eosentomonheatherproctorae*, which is known only from its type locality at the Queen’s University Biological Station near Kingston, Ontario. Thus, the known Canadian fauna totals nine species, eight of which are known only from Canada (Table 2). All nine of these species are reported from Canada in Szeptycki’s comprehensive world catalog (Szeptycki 2007). Although Allen (2006) published a catalog of North American Protura, it was missing a number of the above records, omitted the species *E.heatherproctorae* entirely, contained some apparent typographic errors in relation to the Canadian fauna, and was entirely superseded by Szeptycki (2007).

The Protura fauna of Alaska comprises 15 species (Szeptycki 2007), none of which have yet been reported from Canada, but some of them may occur in adjacent unglaciated parts of the Yukon. Nineteen species are known from US states in close proximity to the southern Canadian border, 17 from Michigan and one each from Idaho and Vermont, and some of these species also may occur in southern Canada. As the interior of Alaska wasn’t glaciated, it isn’t surprising that it has a large Protura fauna. However, in contrast, Michigan was entirely buried under the Laurentide Ice Sheet during the Wisconsin Glacial Period, yet it has almost twice the known species richness of Canada. This indicates that substantial post-glacial recolonization from the south has occurred and that species richness in southern Canada is likely much higher than we know. Of the 32 Protura species known from Alaska, Michigan, Idaho, and Vermont that are not recorded from Canada, it is conservatively estimated that 10 species, five in each family, may occur in Canada. Thus at least 53% of the Canadian fauna remains undocumented.

There are three BINs of Canadian Protura based on specimens collected in Ontario, all identified as family Eosentomidae (BOLD:ACY5591, BOLD:ADA0787, BOLD:ADA0788), and all between 17–20% divergent from their nearest neighbors. These BINs could represent species reported from Canada, but species-level determinations are not yet available.

In summary, the poor state of knowledge about the Canadian (and North American) Diplura and Protura fauna offers many opportunities to explore the diversity, distribution, and biology of these tiny but fascinating creatures. In particular, Berlese, Winkler, and Tullgren funnel extractions of litter and decaying wood will greatly aid the documentation of the Canadian fauna. Those who sample more commonly studied soil and litter creatures, such as mites and Collembola, are well-situated to enhance Canadian collections of other poorly studied litter arthropods by saving by-catch of groups such as Protura and Diplura and forwarding it to those willing to study and identify the material using traditional or molecular methods. Given the difficulty of collecting intact specimens that retain enough appendages to allow morphology-based identification, their small size, and the scarcity of taxonomists interested in diplurans and proturans, it is expected that genetic data will play an increasingly important role in advancing our understanding of the Canadian fauna for these taxa.

**Table 2.** Census of Protura in Canada.

Taxon^1^	No. species reported in Tomlin (1979b)	No. species currently known from Canada	No. BINs^2^ available for Canadian species	Est. no. undescribed or unrecorded species in Canada	General distribution by ecozone^3^	Information sources
**Order Acerentomata**						
Acerentomidae	2	6	0	5	Taiga Cordillera, Pacific Maritime, Hudson Plains	Szeptycki 2007
**Order Eosentomata**						
Eosentomidae	1	3	3	5	Hudson Plains	Szeptycki 2007; BOLD
**Total**	**3**	**9**	**3**	**10**		

^1^Classification follows that of Szeptycki (2007) with one recent update in the text from Shrubovych et al. (2014). ^2^Barcode Index Number, as defined in Ratnasingham and Hebert (2013). **^3^**See figure 1 in Langor (2019) for a map of ecozones.
